# Severe hemodynamic instability during elective surgery for a patient with a giant pheochromocytoma: A case report

**DOI:** 10.1016/j.ijscr.2019.01.021

**Published:** 2019-01-30

**Authors:** J.Y. Hu, C. Wang, H.B. Wang, P.X. Chen, Z.J. Zhen, W.Y. Lau

**Affiliations:** a Department of Hepatopancreas Surgery, The First People’s Hospital of Foshan, Guangdong, China; b Department of Anesthesiology, The First People’s Hospital of Foshan, Guangdong, China; c The First People’s Hospital of Foshan, Guangdong, China; d Faculty of Medicine, The Chinese University of Hong Kong, Shatin, New Territories, Hong Kong Special Administrative Region

**Keywords:** Pheochromocytoma, Surgery, Anaesthesiology, Laparotomy

## Abstract

**Introduction:**

We present a patient with a huge pheochromocytoma who ran a stormy intraoperative course.

**Presentation of case:**

A 57-year-old woman underwent elective open surgery for a giant pheochromocytoma (16 × 15 × 10 cm) after adequate preoperative medical preparation. The patient developed severe hypertension on tumor mobilization followed bylife-threatening hypotensionwhen the tumor was removed. The hemodynamic instability was successfully managed. Histology showed a pheochromocytomawith tumor-free resection margins. The patient fully recoveredandthe hypertension completely resolved after the operation.

**Discussion:**

This is a rare and educational case report on a patient with a huge pheochromocytoma who was successfully managed by a multidisciplinary team of specialists.

**Conclusion:**

Pheochromocytoma should be resected if technically possible. A multidisciplinary team approach is required for proper management.

## Highlights

• A huge pheochromocytoma measuring 16 cm × 15 cm × 10 cm is rare.
• Despite adequate preoperative and intraoperative management, the patient developed severe hypertension followed by a hypotension crisis which were almost lethal.
• Through multidisciplinary management with appropriate resuscitative measures, the huge tumor was resected en bloc with a R0 resection.
• The patient recovered well postoperatively and was free of hypertension.

## 1 Introduction

Intraoperative maintenance of hemodynamic stability in patients with a pheochromocytoma is challenging, or even breathtaking, for the anaesthetists [1,2]. Approximately 15% of patients would present with hemodynamic instability, or crisis, even with adequate preoperative preparation [3]. Pheochromocytoma is an uncommon neuroendocrine tumor capable of producing catecholamines, and it predominantly originates from the adrenal gland [4]. Release of epinephrine and norepinephrine could elicit typical symptoms which include excruciating headache, excessive sweating, hypoglycemia and intermittent or persistent hypertension [5,6]. These symptoms are effectively mitigated after the tumor is removed. However, during surgery to remove the tumor, sudden fluctuations in levels of catecholamines can occur with resultant severe fluctuations in systemic blood pressure, which can be life-threatening. Adequate preoperative medical treatment for heart rate control and adequate volume expansion are required and paramount. Intraoperative anaesthetic managements including careful monitoring of blood pressure and anticipated vasopressor drug support are important to prevent severe hypotension [7,8]. An unusual case of a patient with a giant pheochromocytoma is herein reported. This patient experienced severe hemodynamic instability during elective surgery to resect the tumor.

The work has been reported in line with the SCARE criteria [9].

## 2 Consent for publication

The patients gave her written permission for the authors to publish the report.

## 3 Presentation of case

**Fig. 1:**
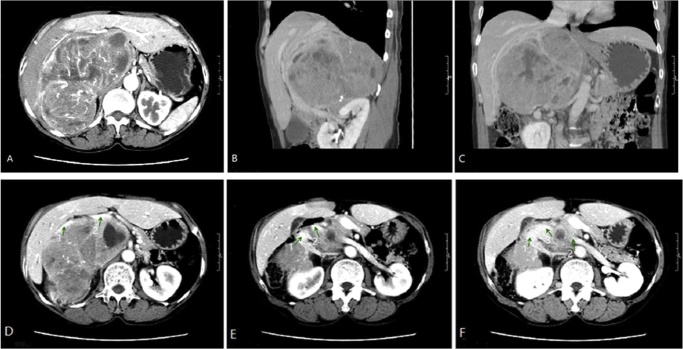
The CT images of the tumor where venous involvement was considered. Fig. 1D: inferior vena cava(arrow 1), portal vein (arrow 2); Fig. 1E: right renal vein(arrow 1), left renal vein(arrow2); Fig. 1F:right renal vein(arrow 1), left renal vein(arrow2), inferior vena cava(arrow 3).

A 57-year-old woman presented to the outpatient department with an intra-abdominal mass. The patient gave a history of hypertension treated with an alpha-blocker(Terasozin) and a calcium antagonist (amlodipine).Physical examination revealed that she had a normal blood pressure(BP) of 126/57 mmHg and a heart rate of 98 bpm. A non-tender spherical mass was palpable in the right upper abdomen. A significantly elevated level of urinary catecholamine and a typical adrenal mass on abdominal CT scan (Fig. 1) led to the diagnosis of a large right pheochromocytoma (16 cm × 15 cm × 10 cm). The right renal vein and the inferior vena cava (IVC) were involved by the tumor on CT scanning (Fig. 1). Electrocardiogram (ECG) showed a sinus rhythm. Echocardiography showed normal ventricular function. Laboratory workup and chest X-ray were normal.

The management of this patient was discussed in a multi-disciplinary team meeting which included specialists from the departments of radiology, general surgery, intensive care, anaesthesiology, cardiology, endocrinology and nursing. The decision was to stabilize her blood pressure with Terasozin and amlodipineat the120-130/65-85 mmHg range, and a beta-blocker was not added since the patient’s heart rate was within the normal range. Surgical resection was planned after 3 days of preoperative medical preparation with maintenance of a persistent hemodynamic stability. The American Society of Anesthesiologists (ASA) physical status was 2.

**Fig. 2:**
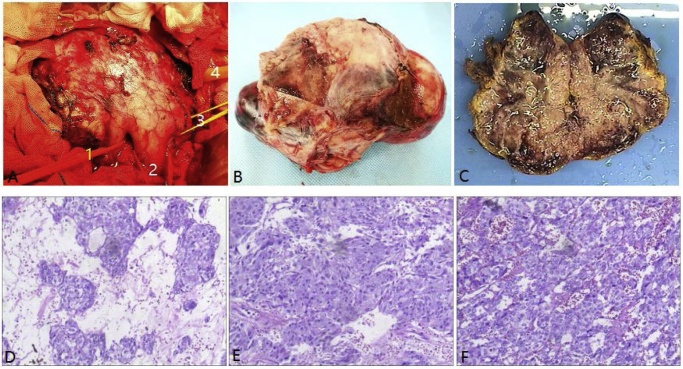
The macroscopic and microscopic images of the tumor. Fig. 3A: right renal vein(1), inferior vena cava(2), left renal vein(3), portal vein(4).

**Fig. 3:**
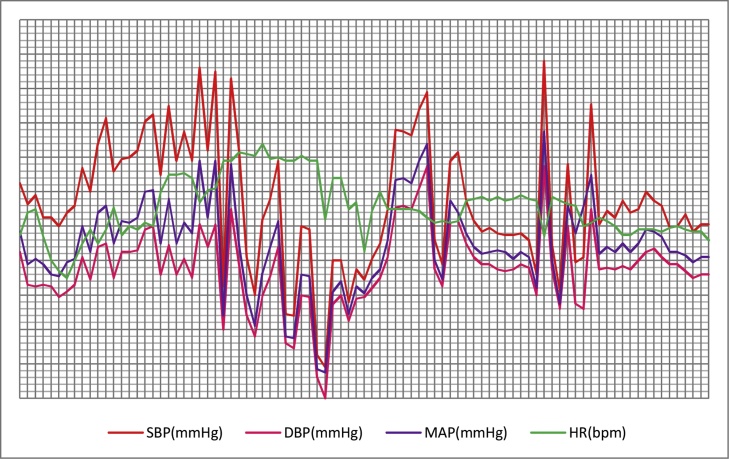
This figure represents the vital signs during surgery: systolic blood pressure (SBP), diastolic blood pressure (DBP), heart rate (bpm).

Before anaesthetic induction, the blood pressure was 122/82 mmHg. After setting up two peripheral intravenous lines, mask ventilation and tracheal intubation followed. When a deep level of anaesthesia was achieved, a central vein pressure(CVP) line for monitoring was conducted via the right internal jugular vein and an arterial blood pressure(ABP) line for continuous monitoring of BP was inserted via the left radial artery. On laparotomy using an inverted “T”-shaped incision, the tumor was found to be completely capsulated. The right renal vein and IVC were compressed by the tumor. Invasion into the surrounding tissues was absent. The tumor was adherent but had not involved the wall of the IVC (Fig. 2).Fluctuations in the BP and heart rate occurred when the tumor was mobilized, with hypertensive peaks reaching to 255/158 mmHg and a heart rate up to 150bpm (Fig. 3). Phentolamine and sodium nitroprusside were used to stabilize the BP at the 70～160/40～100mmHg and HR at 60～120bpmranges (Fig. 3). Severe hypotension occurred when the IVC was blocked to facilitate tumor removal.The patient then developed a hypotensive crisis after the tumor was removed and the IVC was unblocked. The patient recovered following ten minutes of resuscitation by the surgeon performing external cardiac massage and the anaesthetists administering several boluses of epinephrine and norepinephrine, as well as rapid volume expansion. A total of 63 mg of epinephrine and 220 mg of norepineprhine were used. After hemostasis and further stabilization,the patient was transferred to the ICU at the end of the operation. The total intraoperative blood loss was 7300 ml and the operation time was 420 min. After a 3-day stay in the ICU, the patient was transferred to the Hepatopancreatic Surgery Department. Nine days after surgery, the patient was discharged home well. Her hypertension completely resolved after removal of the tumor. On follow-up, the patient fully recovered. Both the urinary and blood catecholamines, aldosterone and cortisol became normalized. The diagnosis of pheochromocytoma was confirmed by histopathology. The resection margins were clear of tumor (R0 resection).

## 4 Discussion

The intraoperative management of patients with a pheochromocytoma canturn out to be a nightmare for the anaesthetists. Operative morbidity and mortality rates have been reported to be as high as 13% in the 1980s [2]. Currently, surgical resection of a pheochromocytoma can still be life-threatening although perioperative management has greatly improved [10]. Pheochromocytoma is capable of producing excessive amounts of catecholamine sresulting in peripheral vascular constriction, reduced blood volume and persisten thypertension [11]. Lethal hypertensive crisis happens when great amounts of catecholaminesare releasedwhen apatient is irritated or when a tumor is pressed [12].

Our patient was diagnosed to have a pheochromocytoma on the basis of hypertension, elevated levels of urinary VMA and an adrenal mass on CT scan. Most reported cases of pheochromocytoma are less than 10 cmin size [13,14]. A benign pheochromocytoma which is under 6 cm in size can be treated laparoscopically [15,16]. In our patient, laparotomy was decided since the tumor was 16 cm in its greatest diameter and the renal vein and IVC were possibly involved.

Other than the uncommon size of the tumor, what makes this case interesting and educational is the severe hemodynamic instability during surgery. Despite careful preoperative preparation and intraoperative anaesthetic management, dramatic fluctuations in the BP and heart rate occurred, with severe hypertension followed by a hypotensive crisis. Such fluctuations have been reported in patients with a pheochromocytoma,but not as dramatic as in our patient. Although the underlying pathophysiological mechanisms of this hemodynamic fluctuation remain unclear [17], the large tumor size with excessive catecholamines secretion during manipulation of the tumor probably led to the hypertensive crisis in our patient. The inadequate volume replacement, the large intraoperativeblood loss (7300 ml) during surgery,and the clamping of the IVC probably resulted in the severe hypotension. Furthermore, persistent high levels of catecholamines in patients with a pheochromocytoma could result in adrenergic receptor insensitivity, with reduced blood volume, blood cell redistribution and damage to the myocardia. After the tumor was removed, acute withdrawal of catecholamines secretion could also induced the severe hypotension following the hypertensive crisis. Severe hypotension during surgery is likely to result in cardiac arrest which can be irreversible in patients with a pheochromocytoma [2], if the patient is not adequately resuscitated. Fortunately, the patient recovered well after cardiac massage, with rapid volume expansion and extensive provision of catecholamines to provide perfusion to the vital organs.

The positive outcome of this patient can be attributed to the close cooperation of the multidisciplinary team of specialists in the preoperative, intraoperative and postoperative periods. Prior to surgery, surgeons worked with a cardiologist and an endocrinologist to confirm the diagnosis, conducted comprehensive evaluation and stabilized the BP. During surgery, the surgeons and the anaesthestists joined hands to combat the hemodynamic instability. Intensive care specialists helped to stabilize the postoperative period of the patient.

In conclusion, an unusual case of a patient with a giant pheochromocytoma was presented who developed life-threatening hemodynamic instability during surgery, despite adequate preoperative medical preparation to stabilize the patient. A multi-disciplinary team management of this patient played an important role in achieving success in this patient.

## Conflicts of interest

No conflicts of interest.

## Sources of funding

No sources of funding for my research.

## Ethical approval

Not applicable. The study is exempt from ethical approval in our institution.

## Consent

Written informed consent was obtained from the patient for publication of this case report and accompanying images. A copy of the written consent is available for review by the Editor-in-Chief of this journal on request

## Author contribution

Lau WY was responsible for study design and reproofing. Zhen ZJ performed the surgery. Wang HB performed the anaesthesia and managed the patient during surgery. Hu JY was an assistant for the surgery. Wang C and Chen PX collected and analyzed tha data as well as wrote this paper. Lau WY and Zhen ZJ were corresponding to this article.

## Registration of research studies

No Needed.

## Guarantor

Hu JY, Wang C, MD, Wang HB, Chen PX, MD,Zhen ZJ, Lau WY.

## Provenance and peer review

Not commissioned, externally peer-reviewed.
